# NAMPT activity plays a key role in driving autoimmune processes that mediate beta-cell death and type 1 diabetes development in mice

**DOI:** 10.1038/s41419-026-08700-6

**Published:** 2026-06-04

**Authors:** Daniel Egbase, Sophie R. Sayers, Naila Haq, Vesela S. Gesheva, Jithu J. Varghese, Sreya Bhattacharya, Jay Kynaston, Ella L. Hubber, Lorna I. F. Smith, Timothy J. Pullen, Henry Gerdes, Vivian K. Lee, David Hopkins, Min Zhao, Yee Cheah, Joshua Greally, Sam Butterworth, James A. Pearson, Gavin A. Bewick, Shanta J. Persaud, Paul W. Caton

**Affiliations:** 1 Department of Diabetes, School of Cardiovascular and Metabolic Medicine & Sciences, Faculty of Life Sciences and Medicine, King’s College London, London, UK; 2 Institute of Diabetes and Regeneration Research, Helmholtz, Munich, Germany; 3 Cell Signalling and Proteomics Group, Centre for Genomics & Computational Biology, Barts Cancer Institute, Queen Mary University of London, Charterhouse Square, London, UK; 4 Division of Pharmacy and Optometry, Faculty of Biology, Medicine and Health, University of Manchester, Manchester, UK; 5 Division of Infection and Immunity, Cardiff School of Medicine, Cardiff University, Cardiff, UK

**Keywords:** Type 1 diabetes, Interferons, Cell death and immune response

## Abstract

Type 1 diabetes (T1D) is characterised by destruction of pancreatic beta-cells by islet-infiltrating cytotoxic lymphocytes and elevated intra-islet secretion of pro-inflammatory cytokines. However, the underlying pathophysiological mechanisms remain incompletely understood. We hypothesised that abnormal elevation of islet NAD, via activation of NAMPT, plays a key role in driving islet autoimmune processes, leading to beta-cell death in T1D. Here, we report that NAMPT inhibition protects against pro-inflammatory cytokine (IL-1β, TNFα and IFNγ) mediated beta-cell dysfunction and apoptosis in isolated mouse and human islets. RNAseq revealed that NAMPT inhibition blocked cytokine-mediated gene expression linked to pro-inflammatory responses and leukocyte migration. In vivo, diabetes was induced in CD1 mice via multiple low-dose streptozotocin (MLDS) injections. MLDS mice were administered the NAMPT inhibitor FK866 (10 mg/kg; IP) or saline equivalent for 16 days. These experiments demonstrated that NAMPT inhibition improved glycaemic control and beta-cell survival and function in MLDS mice. FK866 also reduced proportions of islet-residing TNFα-producing CD4^+^T-cells and F4/80^+^macrophages, proliferation of spleen-derived CD4^+^ and CD8^+^T-cells and proliferation of islet-derived CD4^+^T-cells and F4/80^+^macrophages. Finally, we report that NAMPT inhibition was able to block pro-inflammatory cytokine-mediated migration of cytotoxic CD8^+^T-cells into isolated islets, using an in vitro transwell platform. This data supports a key immunomodulatory role for NAMPT in islet autoimmunity. NAMPT inhibition may be able to prevent beta-cell death and thus represent a novel therapeutic approach for T1D. The effects of increased NAD levels on islet inflammation require in-depth characterisation and caution should be exercised with regard to the use of NAD boosting supplements, particularly in individuals at risk of developing T1D.

## Introduction

Type 1 diabetes (T1D) is an autoimmune disorder characterised by auto-reactive immune cell-mediated death of pancreatic beta-cells [1–4]. The precise triggers that initiate pathogenesis of T1D remain unclear, with both genetic susceptibility and/or environmental triggers reported to prime autoimmune events in affected individuals [5–9]. Autoimmune events in T1D are characterised by islet infiltration by CD4^+^ and CD8^+^T-cells, in a process known as insulitis [1–4, 10]. Cytotoxic CD4^+^ and CD8^+^T-cells secrete pro-inflammatory cytokines (e.g., TNFα, IFNγ) within the intra-islet environment [11–13], causing autoimmune-mediated death of beta-cells.

Several issues surround current T1D treatments, including hypoglycaemia associated with exogenous insulin therapy and poor graft survival following islet transplantation [14–20]. Teplizumab, an anti-CD3 mAb which can delay development of T1D, was recently approved [21]. However, no drugs are currently available which can prevent or treat T1D.

One candidate as a regulator of islet inflammation and T1D drug target is nicotinamide phosphoribosyltransferase (NAMPT). NAMPT is the rate-limiting enzyme in the NAD salvage pathway, where it catalyses conversion of nicotinamide (NAM) to nicotinamide mononucleotide (NMN). NMN is subsequently converted to NAD by NMN adenynyltransferase enzymes [22]. NAD+ metabolism regulates innate and adaptive immune response homeostasis [23]. However, accumulating evidence indicates that NAMPT activity, potentially mediated via enhanced NAD+ levels, can facilitate dysregulation of immunomodulatory processes and onset of inflammation.

For example, NAMPT activity reportedly induces TNFα production in mouse and human inflammatory cells [24] and can promote T-cell activation and IFNγ production in mature T-cells [25–28]. These effects, as well as enhanced T-cell proliferation are reduced following NAMPT inhibition. Interestingly, NAMPT inhibition has no effect on inactive T lymphocytes [29]. NAMPT activity is also required for activation of pro-inflammatory M1 macrophages and NAMPT inhibition downregulates TNFα expression in M1 macrophages and polarizes macrophages towards an anti-inflammatory M2 phenotype [30–32]. Consistent with these findings, a key role for NAMPT activity in pathogenesis of auto-immune conditions including Inflammatory Bowel Disease, arthritis and autoimmune encephalomyelitis has been reported [29, 33–36]. Further support for a role for NAD in mediating inflammation is derived from recent studies reporting that high levels of the NAD-boosting precursors niacin and nicotinamide riboside (NR) can promote inflammation in macrophages and endothelial cells [37, 38].

Crucially, many of the immunomodulatory effects described above are also key pathogenic events in T1D development. This suggests a potential role for NAMPT activity in the pathophysiology of islet autoimmunity and beta-cell death.

To investigate the therapeutic effects of NAMPT inhibition in T1D, we examined the anti-diabetic effects of two separate NAMPT inhibitors, FK866 [39, 40] and compound 17 (C17[41]), in the multiple-low dose streptozotocin (MLDS) T1D model [42, 43] and in isolated mouse and human islets treated with pro-inflammatory cytokines.

## Results

### NAMPT inhibition protects pancreatic islets from pro-inflammatory cytokine-induced dysfunction and apoptosis

**Fig. 1:**
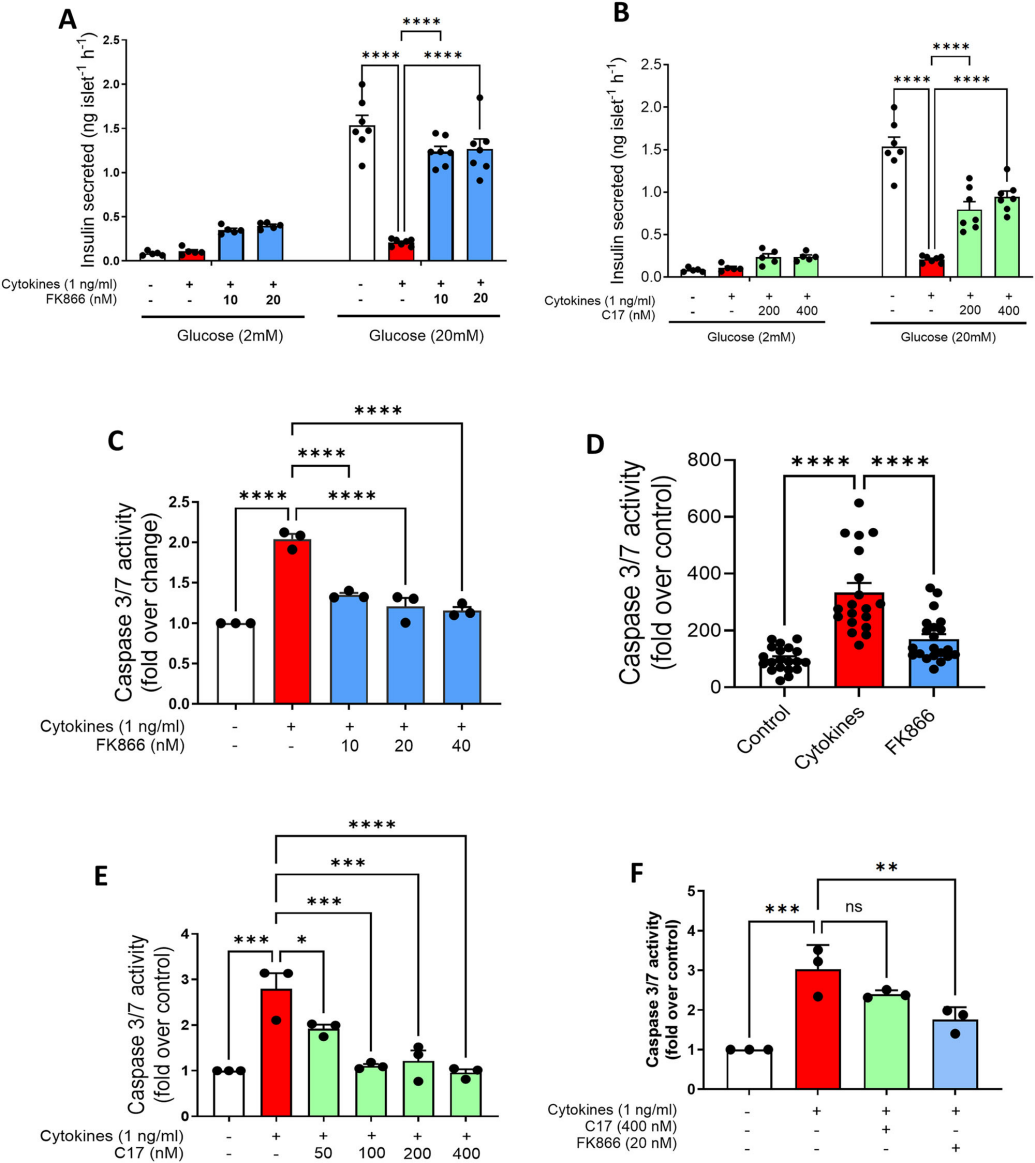
NAMPT inhibition exerts protective effects against pro-inflammatory cytokine-induced beta-cell dysfunction and apoptosis in isolated pancreatic islets. Static insulin secretion in response to basal (2 mM) or 20 mM glucose was assessed in isolated mouse islets incubated with cytokines (CK; IL-1β, TNFα and IFNγ; 1 ng/ml), with or without either **A** FK866 (10–20 nM) or **B** compound 17 (200–400 nM) for 24 h. *N* = 5–7 replicates from 6 mice and representative of three independent experiments. Apoptosis (caspase 3/7 activity) was measured in isolated mouse islets treated with (**C**) CK cocktail with or without FK866 (10–40 nM) for 24 h or **D** in mouse islets pre-treated with FK866 (10 nM) for 2 h followed by CK treatment (without FK866) for 22 h. **E** Apoptosis (caspase 3/7 activity) measured in isolated mouse islets treated with CK cocktail with or without compound 17 (50– 400 nM) for 24 h. For **C**, **E** *n* = 3, where an *n* of 1 is equal to one experiment with 10 wells, each with five size-matched islets. For **D** *n* = 20 where an n of 1 is equal to one experiment with 10 wells, each with five size-matched islets (**F**). Apoptosis (caspase 3/7 activity) was measured in isolated human islets treated with a CK cocktail with or without either FK866 (20 nM) or compound 17 (400 nM) for 24 h. *n* = 3, where an n of 1 is equal to one experiment with 10 wells, each with five size-matched islets. Statistical significance was tested using two-way ANOVA with Tukey’s post-hoc test (**A**, **B**) and one-way ANOVA with Bonferroni’s post-hoc test (**C**–**F**). Values in all graphs are represented as means ± SEM. Statistical significance between groups is depicted by *p < 0.05, **p < 0.01; **p < 0.001 ****p < 0.0001.

Beta-cell dysfunction and death in T1D are partly mediated by exposure to pro-inflammatory cytokines. To determine the importance of NAMPT in beta-cell failure in T1D, we exposed mouse and human islets to pro-inflammatory cytokines ± NAMPT inhibitors (FK866 or C17; Supplementary Fig. 1A–G). Static glucose-stimulated insulin secretion (GSIS) studies showed that both FK886 and C17 exerted protective effects against cytokine-mediated reductions in GSIS (both *p* = <0.0001 vs. Cytokines alone) (Fig. 1A, B). Neither FK866 (10-20 nM) nor C17 exerted effects on static GSIS when added in the absence of cytokines (Supplementary Fig. 2A). Co-incubation and pre-treatment with FK866 (Fig. 1C, D; *p* = <0.0001 vs. Cytokines alone) or C17 (Fig. 1E) also significantly protected against apoptosis (caspase 3/7 activity) in mouse islets. Finally, we observed that both FK866 and C17 also prevented cytokine-mediated apoptosis in human islets (Fig. 1F). FK866 had no effect on caspase 3/7 activity in the absence of cytokines (Supplementary Fig. 2B).

### FK866 protects pancreatic mouse islets from deleterious pro-inflammatory cytokine-mediated transcriptional changes

**Fig. 2:**
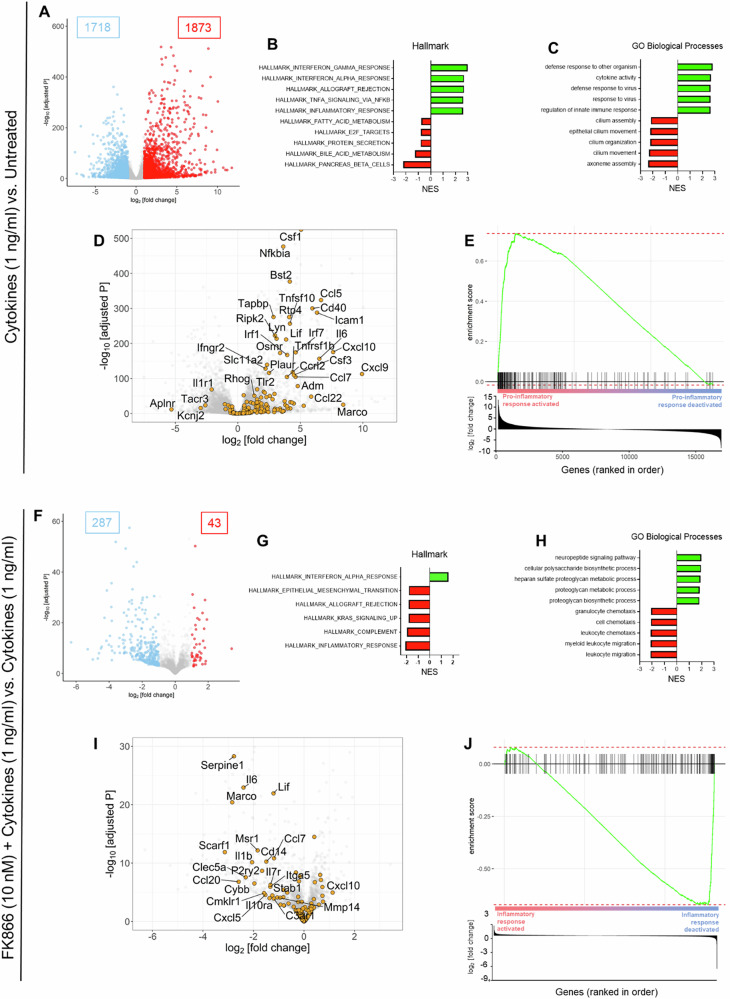
FK866 suppresses cytokine-induced inflammatory transcriptional pathway enrichment in isolated pancreatic mouse islets. Volcano plot depicting log2 fold changes of differentially expressed (DE) annotated genes from RNA-seq performed on isolated mouse islets treated for 24 h with **A** a cytokine cocktail (IL-1β, TNFα and IFNγ; 1 ng/ml) versus untreated or **F** FK866 (10 nM) and a cytokine cocktail versus a cytokine cocktail. Each dot represents a gene. DE genes with a log2 fold change of >1, all adjusted *P* < 0.05 are highlighted in red (indicating upregulation) or blue (indicating downregulation). Numbers denote the total number of upregulated and downregulated genes between treatment groups. **B**, **C**, **G**, **H** Five most and least enriched pathways as revealed by gene set enrichment analysis (GSEA) in isolated mouse islets treated for 24 h with **B**, **C** a CK cocktail versus untreated or **G**, **H** FK866 (10 nM) and a cytokine cocktail versus a cytokine cocktail. GSEA performed with **C**, **G** ‘Hallmark’ and **B**, **H** ‘Gene Ontology (GO) biological processes’ gene sets from the Molecular Signatures Database (MSigDB). Only gene sets with significant (*p* < 0.05) normalised enrichment scores are shown. Volcano plot depicting log2 fold changes of DE genes within the ‘HALLMARK_INFLAMMATORY_RESPONSE’ gene set. The annotated genes are from RNA-seq performed on isolated mouse islets treated for 24 h with **D** a CK cocktail versus untreated or **I** FK866 (10 nM) and a cytokine cocktail versus a cytokine cocktail. Each dot represents a gene. Gene names are displayed for top differentially expressed genes (log2 fold change >1, adjusted *P* < 0.05). Enrichment plots derived from the GSEA performed on the transcripts within the ‘HALLMARK_INFLAMMATORY_RESPONSE’ gene set in isolated mouse islets treated for 24 h with **E** a CK cocktail versus untreated or **J** FK866 (10 nM) and a cytokine cocktail versus a cytokine cocktail. The green line denotes the running statistic, and the black lines represent a single gene in the gene set. Bottom left figures are bar plots showing all DE genes' log2 fold changes ranked in order. *n* = 3 independent biological sample replicates (each replicate consisted of 150 islets).

To understand the effect of NAMPT activity/inhibition in greater detail, we conducted transcriptome analysis on isolated mouse islets co-treated with cytokines and FK866 using bulk RNA sequencing (RNA-seq). Cytokine treatment significantly upregulated 1873 genes and downregulated 1718 genes (Fig. 2A). Gene set enrichment analysis (GSEA) revealed that the most elevated responses were downstream of IFNγ and TNF*α* cytokine activity). Key beta-cell functional and maturity genes were downregulated (Fig. 2B, C). More in-depth analysis of ‘Inflammatory response‘ gene sets illustrates that many of the cytokine-induced upregulated genes encode for proteins mediating or responding to inflammation and cellular stress. These include chemokines (*Ccl5*, *Cxcl10*, *Cxcl9*), cytokine receptor proteins (*Tnfsf10*, *Tnfrsf1b*, *Osmr*) and antigen-processing and -presenting proteins (*Tapbp, Cd40, Marco*), indicative of islet cells experiencing a pro-inflammatory response (Fig. 2D, E).

Compared to cytokines alone, FK866 + cytokine-treated islets had 43 and 287 upregulated and downregulated genes, respectively (Fig. 2F). Notably, FK866 induced downregulation of pathways related to leukocyte migration and chemotaxis and granulocyte chemotaxis. Interestingly, FK866 reversed cytokine-induced upregulation of the ‘Inflammatory response’ gene set (Fig. 2G, H), including chemokines and chemokine receptors (*Ccl20*, *Cxcl5*, *Cmklr1*), cytokine receptors (*Il10r*, *Il7r*, *Osmr*), cytokines (*Il1b*, *Il6* and *Lif*) and innate immune response genes (*Clec5a*, *Cybb*) (Fig. 2I, J).

To gain additional insight into the underlying mechanisms driving these effects, we examined the effects of pro-inflammatory cytokines and NAMPT inhibition on a custom NAD metabolome gene set (Fig. S3). Pro-inflammatory cytokine treatment significantly increased expression of NAMPT (2.62-fold increase), CD38 (2.9-fold increase) and CD157 (6.5-fold increase) following islet pro-inflammatory cytokine treatment. NAMPT inhibition prevented cytokine-mediated increases in NAMPT, CD38 and CD157 gene expression. CD38 and CD157 promote NAD degradation, leading to the generation of NAADP and cADPR, which in turn can induce macrophage and T-cell differentiation and activation. These changes in CD38 and CD157 provide a plausible explanation for the changes in inflammation observed here. Sirtuins are also dependent on NAMPT and NAD. Sirtuin gene expression levels were assessed using our RNAseq data set. SIRT1 and SIRT2 levels were significantly increased in cytokine-treated islets, whereas SIRT3, 4 and 5 levels were decreased in response to cytokines. NAMPT inhibition prevented cytokine-mediated increases in SIRT1/2 and prevented cytokine-mediated decreases in SIRT3,4 and 5 (Fig. S3). We did not observe any significant changes in SIRT6 and SIRT7.

### NAMPT inhibition ameliorates hyperglycaemia and reduces insulin content in MLDS-induced diabetic mice

**Fig. 3:**
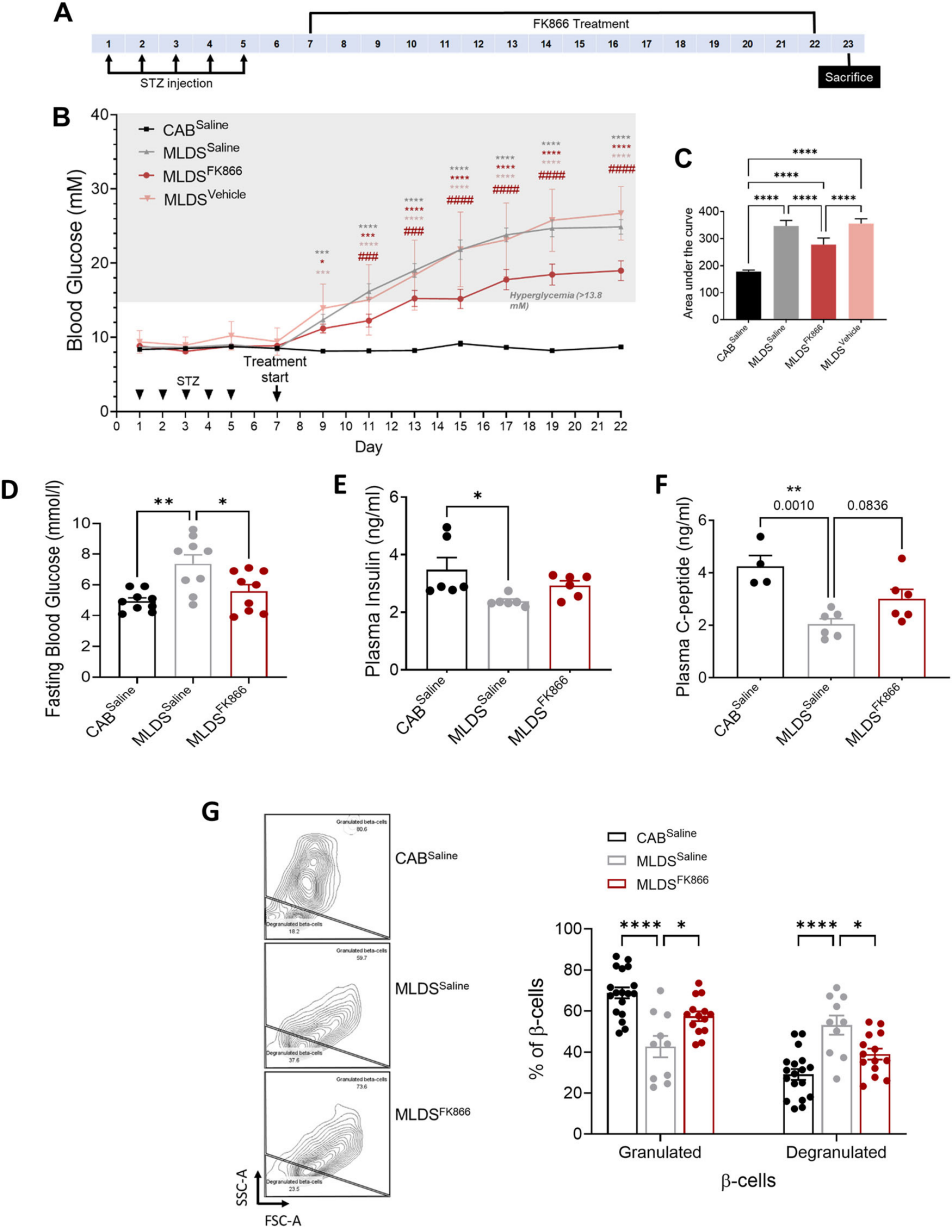
FK866 ameliorates MLDS-induced hyperglycaemia and reduced beta-cell functionality. **A** Schematic diagram of the experimental protocol: mice were administered citric acid buffer (CAB) or multiple low-dose streptozotocin (MLDS) regimen, then after 48 h, were administered saline, FK866 or vehicle (IP) for 17 days. **B** Ambient blood glucose was measured every 2–3 days and **C** the corresponding area under the curve was analysed. In **B**, black arrows and annotations denote streptozotocin (STZ) exposure and FK866 treatment (*n* = 20). **D** Fasting blood glucose (day 23; *n* = 9); **E**, **F** Plasma levels of insulin and C-peptide measured by ELISA using terminal blood samples taken on the day of sacrifice (day 23) following fasting and then injection of 2 g/kg 30% glucose solution (*n* = 6). For **B**–**F**, n of 1 is equal to one mouse. **G** Representative image of data showing SSC/FCS of granulated and degranulated β cells on the day of sacrifice (day 23). Also shown are corresponding percentages of the optical properties of insulin-positive islet cells following isolation, dispersion and immunofluorescence staining of insulin. *n* = 14–18 pools of islet cells of which a maximum of 5000 cells were analysed. Statistical significance was tested using two-way ANOVA with Tukey’s post-hoc test (**B**, **G**) and one-way ANOVA with Bonferroni’s post-hoc test (**C**–**F**). Values in all graphs are represented as means ± SEM. Statistical significance vs CAB^Saline^ is depicted by *p < 0.05, **p < 0.01, ***p < 0.001, ****p < 0.0001. Statistical significance vs MLDS^Saline^ is depicted by #*p* < 0.05, ##*p* < 0.01, ###*p* < 0.001, ####*p* < 0.0001.

Given that NAMPT activity played a role in cytokine-induced beta-cell survival and function in vitro, we next sought to assess the effects of NAMPT inhibition with FK866 on the course of diabetes development in vivo. Diabetes was induced in mice by multiple-low dose streptozotocin (MLDS) administration (40 mg/kg bw; IP; 5 consecutive days). FK866 was administered to MLDS mice, beginning at day 7 (two days post end of STZ administration; see Fig. 3A). From day 11 until the end of the experiment (day 23), FK866 treatment significantly lowered ambient (Fig. 3B, C) and fasting (Fig. 3D) blood glucose concentrations in MLDS^FK866^ mice, compared to MLDS^Saline^ and MLDS^Vehicle^ treatment.

To determine the effects of NAMPT inhibition on beta-cell function, we assessed whether FK866 enhanced serum insulin and C-peptide response to glucose. MLDS^Saline^ mice exhibited significantly lower plasma insulin (*p* = 0.05) and c-peptide (*p* = 0.01) compared to non-diabetic/CAB^saline^ animals. FK866 treatment slightly, but non-significantly, prevented MLDS-mediated reductions in insulin (Fig. 3E) and c-peptide secretion (3F, *p* < 0.08). Beta-cell health was examined in greater detail through flow cytometry assessment of insulin content. MLDS induced a significant reduction in the sub-population of granulated beta-cells (*p* = 0.0001) and a significant increase in sub-populations of degranulated beta-cells (*p* = 0.0001). Consistent with a beta-cell protective effect, FK866 significantly increased granulated beta-cell proportion (*p* < 0.05) and decreased proportion of degranulated beta-cells (*p* < 0.05) compared to the MLDS^Saline^ (Fig. 3G). Interestingly, we did not observe any changes across all three groups in liver NAD and NADH levels, NAD/NADH ratio or liver histone H3 acetylation status (a marker of sirtuin activity) (Fig. S4). This is an important observation with regard to the clinical utility of NAMPT inhibitors, indicating that administration may target islets/pancreas selectively in this model and not lead to off-target (i.e. non-pancreatic) effects.

Overall, these findings suggest that NAMPT inhibition with FK866 improves glycaemic control, in part through partial protection of beta-cell function and maintenance of beta-cell insulin content during MLDS-induced diabetes development.

### NAMPT inhibition protects MLDS-induced diabetic mice from insulitis

**Fig. 4:**
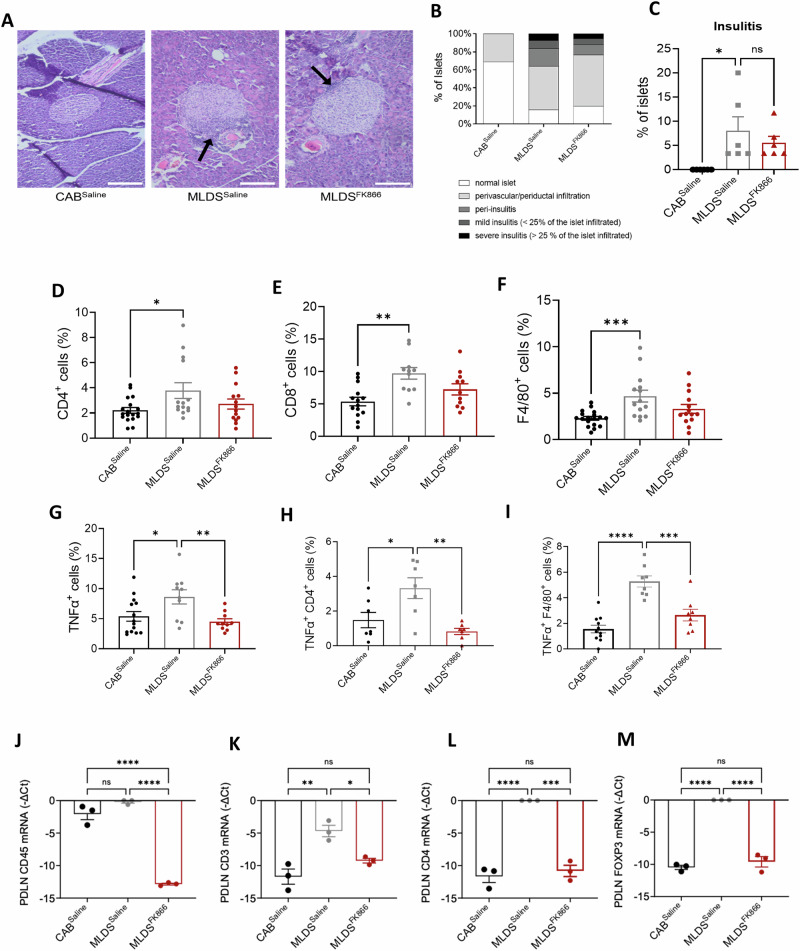
FK866 partially protects MLDS-induced diabetic mice from insulitis. **A** Representative images of mouse pancreas from CAB^Saline^, MLDS^Saline^ and MLDS^FK866^ mice stained with hematoxylin and eosin and evaluated for severity of infiltrating immune cells using light microscopy. Black arrows indicate immune cell infiltration. Imaged at 20× (Scale bars, 50 μM). **B** Corresponding percentages of islets graded for all stages of insulitis. **C** Percentages of islets showing mild and severe insulitis only. Data generated from 60 islets per mouse pancreas (*n* = 6 mice per group). Percentage of **D** CD4^+^, **E** CD8^+^ and **F** F4/80^+^ cells within the islet cell population of CAB^Saline^, MLDS^Saline^ and MLDS^FK866^ mice following sacrifice. *n* = 14–18 pools of islets of which a maximum of 5000 cells were analysed. Percentages of **G** TNFα + , **H** TNFα + CD4 + , **I** TNFα + F4/80 + , within the islet cell of CAB^Saline^, MLDS^Saline^ and MLDS^FK866^ mice. *n* = 7–14 pools of islets of which a maximum of 5000 cells were analysed. Gene expression of immune cell markers in PDLN isolated from CAB^Saline^, MLDS^Saline^ and MLDS^FK866^ mice following sacrifice; mRNA levels of **J** CD45, **K** CD3, **L** CD4 and **M** FOXP3 were assessed by qPCR (*n* = 3). Statistical significance was tested using one-way ANOVA with Bonferroni’s post-hoc test. Values in all graphs are represented as means ± SEM. Statistical significance is depicted by *p < 0.05, **p < 0.01, ***p < 0.001, ****p < 0.0001.

We next sought to determine whether the effects of FK866 on glycaemic control and beta-cell health were linked to suppressed insulitis. Whilst all severities of insulitis were observed in MLDS^Saline^ and MLDS^FK866^ mice, MLDS^FK866^ mice exhibited a greater proportion of islets that remained in the perivascular/periductal infiltration stage rather than progressing to the peri-insulitis/insulitis phase (Fig. 4A-C), indicating partial protection against insulitis by FK866. To gain a deeper insight into these effects, the proportion of immune cell subtypes present within the islet was examined using flow cytometry. MLDS^Saline^ mice displayed a ~2-fold increase in islet CD4^+^T-cells (*p* = 0.05), CD8^+^T-cells (*p* < 0.01) and F4/80^+^macrophages (*p* < 0.001), compared to non-diabetic controls (Fig. 4D–F). Similar to the H + E data, FK866 administration led to a partial decrease (~25%) in the proportion of islet CD4^+^T-cells, CD8^+^T-cells and F4/80^+^macrophages, compared to MLDS^Saline^ (Fig. 4D–F). A crucial factor in immune-mediated T1D pathophysiology is activation of immune cells leading to TNFα and IFNγ production and consequent beta-cell death. MLDS^Saline^ mice had significantly elevated proportions of TNF*α*^+^ cells in the total islet population compared to non-diabetic mice (*p* = 0.05) and FK866 blocked this effect (Fig. 4G; *P* < 0.01). Assessment of immune sub-populations within all TNF*α*^+^ cells revealed MLDS^Saline^ mice displayed significantly increased proportions of TNF*α*^+^CD4^+^ (*P* < 0.05) and TNF*α*^+^F4/80^+^ cells (*P* < 0.0001) compared to non-diabetic control mice and these increases were blocked by FK866 (*p* = 0.01 and *p* = 0.001, respectively; Figs. 4H, I). PDLN analysis of immune cell marker gene expression in PDLNs revealed significantly elevated mRNA levels of CD3 (total T-cells; *P* < 0.01), CD4 (*P* < 0.0001) and FoxP3 (Treg cells; *P* < 0.0001) in MLDS^Saline^ mice compared to control. In all cases, increased expression of immune cell markers was prevented by FK866 administration. (Fig. 4J–M). MLDS^Saline^ mice also displayed significantly elevated proportions of islet IFNγ^+^ cells (*p* < 0.05; Supplementary Fig. 12) and partial increases in IFNγ^+^CD4^+^ and IFNγ ^+^F4/80^+^ sub-populations (Supplementary Fig. 12) FK866 administration led to small but non-significant reductions in these effects. TNF*α*^+^CD8^+^ and IFNγ^+^CD8^+^ cell populations, as well as PDLN CD8 mRNA levels were unchanged across all groups. Together this data demonstrates that NAMPT inhibition can prevent islet immune-cell activation, specifically by reducing TNFα production from islet CD4^+^T-cells and F4/80^+^macrophages. This provides a plausible mechanism through which FK866 prevents beta-cell death and improves glycaemic control in MLDS mice.

### NAMPT inhibition reduces splenic- and islet-derived immune cell proliferation in MLDS-induced diabetic mice

**Fig. 5:**
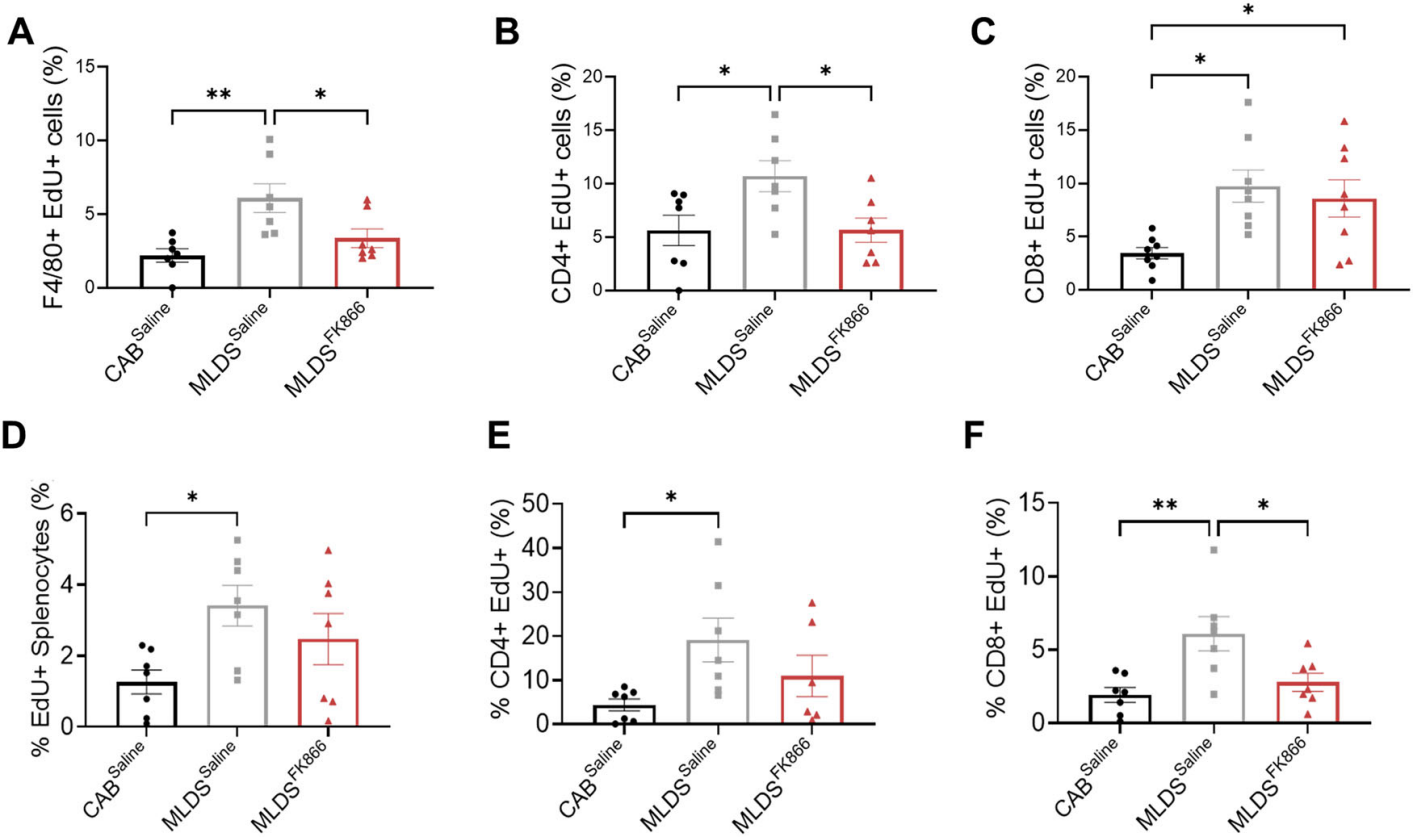
FK866 blocks MLDS-mediated increased proliferation of islet- and spleen-derived immune cells. Percentages of **A** F4/80^+^EdU^+^, **B** CD4^+^EdU^+^, **C** CD8^+^EdU^+^ cells within the islet cell population of CAB^Saline^, MLDS^Saline^ and MLDS^FK866^ mice following sacrifice, pancreatic islet isolation, dispersion, EdU-supplemented culture (72 h) staining and flow cytometric analysis. Percentages of **D** EdU^+^ splenocytes, **E** CD4^+^EdU^+^, **F** CD8^+^EdU^+^ cells within the splenocyte population of CAB^Saline^, MLDS^Saline^ and MLDS^FK866^ mice following sacrifice, spleen removal, dispersion, culture with EdU (24 h), staining and flow cytometric analysis. *n* = 8 pools of islet or spleen cells of which a maximum of 5000 cells were analysed. Statistical significance was tested using one-way ANOVA with Bonferroni’s post-hoc test. Values in all graphs are represented as means ± SEM. Statistical significance is depicted by *p < 0.05, **p < 0.01.

To further elucidate the immunomodulatory impact of NAMPT inhibition, we examined the effects of FK866 on proliferation of islet-derived CD4^+^ and CD8^+^ T-cells and F4/80^+^ macrophages. MLDS^Saline^ mice displayed significantly increased proportions of proliferating CD4^+^, CD8^+^ and F4/80^+^ cells compared to non-diabetic mice (*p* < 0.01, *p* < 0.05 and *p* < 0.05, respectively) (Fig. 5A–C). FK866 prevented MLDS-induced increases in islet-derived CD4^+^T-cells and F4/80^+^ cell proliferation (Fig. 5A, B; *p* < 0.05), but not CD8^+^T-cells proliferation (Fig. 5C). FK866 also partially prevented MLDS-induced increases (*p* < 0.05) in spontaneous splenocyte proliferation (Fig. 5D). FK866 also prevented MLDS-mediated increases in spontaneous proliferation of spleen-derived CD4^+^ and CD8^+^T-cells (*P* < 0.05), (Fig. 5E, F). Together, these results demonstrate the clear anti-inflammatory, immunomodulatory effect of NAMPT inhibition on CD4^+^ and F4/80^+^ immune cells activation during T1D development.

### NAMPT inhibition prevents cytotoxic CD8^+^T-cell migration into pancreatic islets

**Fig. 6:**
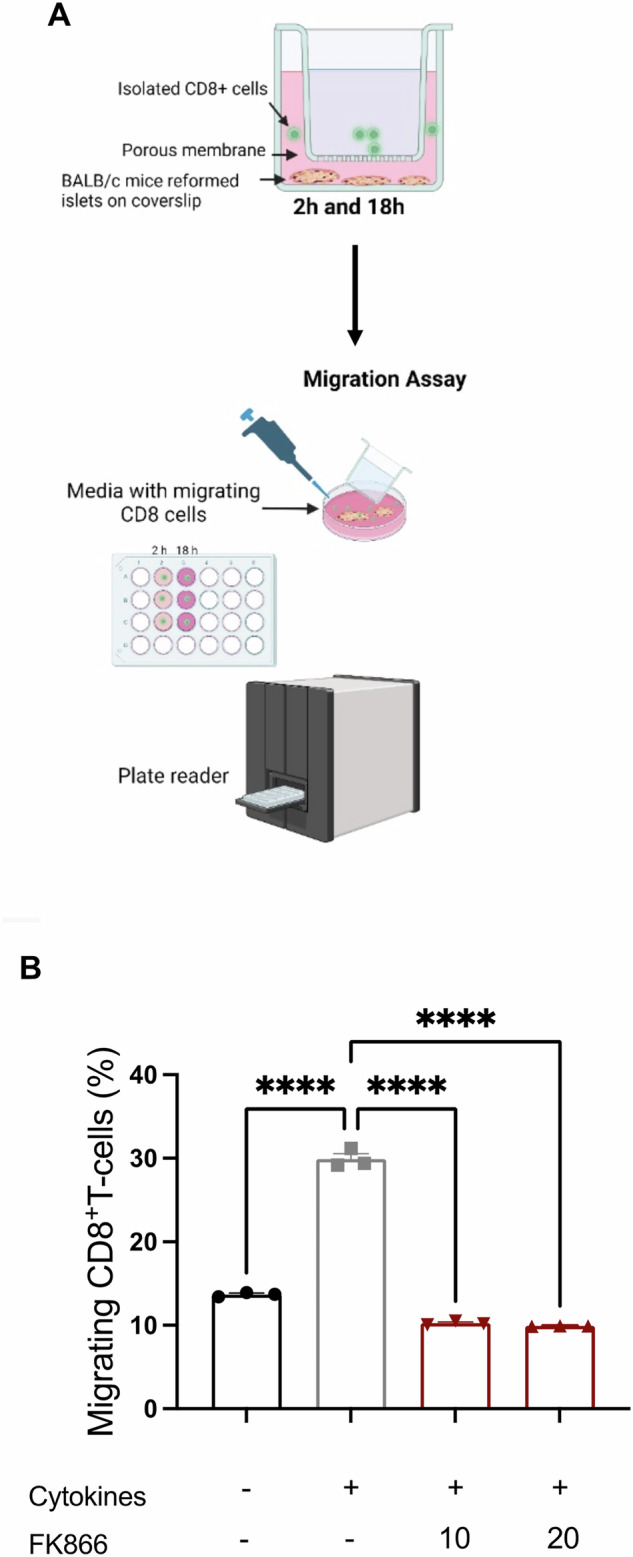
FK866 prevents migration of cytotoxic CD8^+^T-cells into pancreatic islets. **A** Schematic diagram of the experimental protocol. Islets were isolated from BALB/c mice and dispersed and reaggregated into reformed islets. CD8 + T-cells were isolated from splenocytes derived from NY8.3 NOD mice. **B** Islets were exposed to pro-inflammatory cytokines (IL-1β, TNFα and IFNγ; 1 ng/ml), with or without FK866 (10–20 nM) for 18 h. Migration of CD8+ cells into islets was assessed using a trans-well culture platform (*n* = 3). Statistical significance was tested using one-way ANOVA with Bonferroni’s post-hoc test. Values in all graphs are represented as means ± SEM. Statistical significance is depicted by ****p < 0.0001.

To further examine the immunoregulatory effects of FK866, we utilised a transwell culture platform to assess the effect of FK866 on migration of CD8^+^T-cells obtained from NOD mice, into pancreatic islets (Fig. 6A). Exposure of islets to pro-inflammatory cytokines alone led to a 3-fold increase in CD8 + T-cell migration compared to untreated islets (*p* < 0.0001). However, when islets were either pre-treated with FK866 prior to cytokine exposure or co-incubated with FK866 and cytokines, these effects were completely blocked, with CD8 + T-cell migration remaining at basal levels (*p* < 0.0001) (Fig. 6B).

## Discussion

Here, we provide evidence supporting a key immunomodulatory role for NAMPT in T1D pathogenesis. Of potential clinical importance, NAMPT inhibition exerts immunomodulatory effects leading to prevention of cytokine-mediated beta-cell death in isolated islets and improved glycaemic control in MLDS-induced diabetic mice.

Clinical studies report that T1D patients with stimulated plasma C-peptide levels of ≥0.02 pmol/mL exhibit improved metabolic control and lower insulin requirements, compared to T1D patients with plasma C-peptide levels below this threshold [44, 45]. This suggests that even small improvements in beta-cell function, as observed in this study, can lead to clinically relevant improvements in glycemic control in T1D.

These beneficial effects are driven in part by prevention of insulitis and islet immune cell activation. In T1D, auto-immune beta-cell destruction is attributed predominately to increased islet levels of pro-inflammatory cytokines [46, 47]. NAMPT inhibition reduced MLDS-induced IFNγ and TNFα cytokine production in islet cells. This is consistent with previous reports demonstrating that dysregulated NAD+ metabolism is essential for CD4^+^T-cell IFNγ and TNFα secretion [34]. FK866 also prevented migration of CD8^+^T-cells into pancreatic islets. Cytotoxic CD8^+^T-cells are key drivers of T1D pathophysiology and the ability of FK866 to block migration of these cells into islets indicates therapeutic utility of this compound. Transcriptional analysis suggested that NAMPT activity plays a key role in cytokine-induced leukocyte migration and chemotaxis and TNFA- and IFNG-related pathways, providing a potential explanation of the prohibitive effects on insulitis observed in response to NAMPT inhibition in vivo. Interestingly, mRNA levels of Foxp3 (Treg cells) were also elevated in MLDS mice and this effect was prevented following FK866 administration. Potentially, elevated Foxp3 in MLDS mice reflects an attempt to exert anti-inflammatory affects and restore immune tolerance. In contrast, reductions in FoxP3 in FK866 treated mice may reflect overall reduced islet inflammation. Further in-depth characterisation of Treg function and ratios of T-regs to cytotoxic T-cells are required to fully understand these findings.

Importantly, we did not observe any changes in total NAD or NAD/NADH ratio (nor histone acetylation status) in the livers of FK866 treated mice. This indicates that FK866 treatment did not lower NAD levels in other key metabolic tissues, instead potentially targeting islets/pancreas selectively in this model. This is an important observation with regard to clinical utility of NAMPT inhibitors, indicating that administration may not lead to off-target (i.e. non-pancreatic) effects.

Consistent with previous MLDS studies [42, 43], spontaneous proliferation of islet-derived T-cells and macrophages was increased in our model, an effect that was decreased by FK866. This is consistent with studies reporting FK866-mediated inhibition of T-cell proliferation and survival in vitro [31]. NAD can also be metabolised by CD38 and CD157 to generate cADPR and NAADP, which can activate Ca^2+^ signalling pathways critical for T-cell proliferation [48, 49]. Our RNAseq data supports this notion, showing that pro-inflammatory cytokine exposure increases expression of CD38 and CD157 and that this effect of cytokines is blocked by FK866 (Fig. S3).Thus, FK866 may prevent increased CD4^+^T-cell proliferation through indirectly reducing Ca^2+^ availability. Similarly, high levels of the NAD precursors niacin and NR have been reported to promote inflammation via CD38 activation and generation of metabolites 2PY and 4PY [37, 38] and FK866 may impact islet inflammation by blocking these pathways. Separately, other studies investigating the role of NAMPT in macrophages have shown that inflammatory macrophage activation leads to initial NAD depletion, followed by compensatory rises in NAMPT to restore NAD levels. Macrophages consequently become dependent NAMPT for survival and maintenance of inflammatory function [31]. One or a combination of these mechanisms may be responsible for the effects observed in our study and further studies will investigate this in depth.

Our data showed that FK866 protected against cytokine-induced reductions in GSIS in isolated mouse islets. Some previous studies report that NMN administration protected isolated mouse islets from cytokine-attenuated GSIS [50–53]. However, these studies typically utilised levels of NMN (∼100 μM) several times greater than are observed physiologically. Therefore, it is difficult to compare the findings of these experiments with the current study, in which islet NMN levels were generally <10 μM across all treatment groups. In contrast, other studies have reported pro-inflammatory effects of NAD boosting supplements. Although data in islets is currently lacking, NR has been reported to exert pro-inflammatory effects in models of atherosclerosis [37], whilst there are numerous studies reporting pathophysiological effects of NAMPT in other autoimmune diseases, including rheumatoid arthritis, IBD, systemic lupus, psoriasis and autoimmune encephalomyelitis [29, 33–36] which provide support for the findings in T1D presented here.

We report that NAMPT inhibition protects isolated islets from cytokine-induced apoptosis. At first glance, these results are surprising as treating cancer cells with FK866 induces apoptosis following increased caspases-3/-7 activation [54]. However, a recent study found that FK866 supressed caspase-3 activity in H_2_O_2_ stimulated-HeLa cells, but increased LDH release (a measure of necrosis) [55]. The reasoning behind these findings is that as NAMPT activity regulates NAD + and subsequently ATP levels; and because ATP fuels caspase-3 activation, NAMPT therefore plays a key role in determining whether cells undergo caspase-3-mediated apoptosis or caspase-3-independent necrosis. For pancreatic beta-cells, changes in cytokine signalling, NAD+ salvage and intracellular ATP levels could independently or concurrently factor into whether they survive or die. Thus, there may be an ideal, pharmacological level of NAMPT inhibition in pancreatic beta-cells that prevents of extrinsic apoptosis pathway yet does not trigger necrosis. Further investigations are necessary to elucidate the precise mechanisms linking intracellular NAD+ metabolism and beta-cell survival.

## Conclusion

In summary, we provide proof-of-concept evidence identifying NAMPT inhibition as a novel therapeutic intervention to prevent autoimmune beta-cell death during T1D development.

Further research is required to determine the effects of increased NAD levels on islet inflammation and caution should be exercised with regard to use of NAD boosting supplements, particularly in individuals at risk of developing T1D.

## Materials and methods

### MIN6 cell culture

MIN6 cells were cultured at 37 °C in DMEM (25 mmol/l glucose; 2 mmol/l glutamine, 10% FBS (vol./vol.), 100 U/ml penicillin, 100 μg/ml streptomycin) and treated with FK866 or C17 prior to NAD and NMN measurements.

### NMN and NAD+ measurement

NMN was measured in a cell-free environment or in MIN6 β-cells using a previously described fluorometric assay [56, 57]. NAD levels were measured by an NAD/NADH Quantification Kit, (Sigma-Aldrich). See ‘Supplementary methods’.

### Acetylation assay

Acetylation status was measured in liver tissue using a histone H3 acetylation assay kit (Abcam, US; ab115102) according to manufacturer’s instructions.

### Pancreatic islet isolation and treatment

Mouse islets were isolated from 8-week-old non-diabetic CD1 mice, as previously described [58]. Human islets were isolated from heart-beating non-diabetic donors [59] at the King’s College Hospital Human Islet Isolation Unit, with the appropriate ethical approval (KCL HI-RTB; 20/SW/0074; approved by Central Bristol NHS Research Ethics Committee). Informed consent was obtained from all participants. Isolated islets were maintained in supplemented RPMI (37 °C; 5% CO_2_) in a humidified atmosphere for up to 24 h prior to experimental treatment. For insulin secretion and apoptosis measurements ~60 mouse islets were incubated with pro-inflammatory cytokines (1 U/ml Il-1β, 1000 U/ml TNFα and IFNγ) ± either FK866 (10–40 nM) or C17 (50–400 nM) or with FK866 (10 nM)/C17 (200 nM) alone, for 24 h. Mouse islet isolation procedures were performed in accordance with UK Home Office regulations (Animal Scientific Procedures Act, 1986).

### Glucose-stimulated insulin secretion

Mouse CD1 or human islets were pre-incubated in physiological salt solution containing 2 mM glucose. Groups of 5 size-matched islets were then further incubated at 37 °C for 1 h in salt solution and 2 or 20 mmol/l glucose. Secreted insulin was measured using an in-house I125 radioimmunoassay [59].

### Islet apoptosis

Apoptosis was determined using a Caspase-Glo 3/7 luminescent assay (Promega, Southampton, UK).

### Bulk RNA sequencing analysis

Total RNA was extracted from ∼150 islets/mouse (RNeasy Mini Kit, Qiagen, UK). RNA concentration and purity was quantified by NanoDrop (ND-1000, Thermo Fisher Scientific, Hemel Hempstead, UK) spectrophotometer. RNAseq libraries were prepared from 1000 ng total RNA using a NEBNext Ultra II Directional RNA Library Prep kit (New England Biolabs, Hertfordshire, UK). Briefly, mRNA was isolated using the Poly-A mRNA magnetic isolation module (New England Biolabs, Hertfordshire, UK). Isolated mRNA was enzymatically fragmented prior to cDNA synthesis. Each sample was then tagged with a single index that was unique to the sample. cDNA libraries were amplified by PCR for 12 cycles. The resulting library was purified using Agencourt AMpure XP beads (Beckman Coulter, Buckinghamshire, UK). Library quality was assessed using a Bioanlyzer 2100 and DNA High Sensitivity kit (Agilent). Library fragment size distribution was determined using Agilent 2100 expert software. Library concentration was determined by qPCR using NEBNext Library Quant Kit for Illumina (New England Biolabs, Hertfordshire, UK). Libraries were pooled at equal molar ratio and sequenced using an Illumina NovaSeq 6000 at 150 bp paired end with an approximated depth of 25 million reads per sample.

### Diabetic mouse models

Diabetes was induced by multiple-low dose streptozotocin (MLDS) administration [42, 43]. 4–6-week-old CD1 mice (26–33 g) (Charles River, UK) were randomly assigned into four groups: non-diabetic control (citric acid buffer (CAB)^Saline^), diabetic control (MLDS^Saline^), diabetic FK866-treated (MLDS^FK866^) and diabetic vehicle-treated (5% in DMSO in saline; MLDS^Vehicle^). To induce diabetes, mice were administered 40 mg/kg/body weight STZ (IP; Bio-techne, Bristol, UK; dissolved in CAB, pH 4.5) or CAB alone, for 5 consecutive days (days 1–5). FK866 (10 mg/kg body weight; IP) or vehicle control were administered daily (16 days; Days 7–22). Mice were housed in 12 h light/dark cycle, temperature-controlled conditions with *ad libitum* access to standard mouse chow and water. Procedures were performed in accordance with UK Home Office regulations (Animal Scientific Procedures Act, 1986). Ethical approval for all procedures was provided by UK Home Office Project Licence PP9071010 (Improving therapies for diabetes).

### Diabetes evaluation

Ambient blood glucose levels were measured every 2–3 days at 10 am and diabetes was defined by blood glucose levels >13.8 mM over 3 consecutive days. Blood samples were taken from the tail tip. Capillary blood glucose levels (mM) were determined using an Accucheck glucose meter (Roche Diagnostics, UK). Plasma insulin (Mercodia, Sweden) and C-peptide (ALPCO, United States) were measured by specific ELISA in blood collected 30 min after 2 g/kg body weight 30% glucose solution (IP) administration, following overnight fast.

### Pancreas histology

Whole pancreas was excised and fixed in 4% paraformaldehyde for 24 h prior to tissue processing (Leica TP 1020, Leica Biosystems, Germany) and paraffin embedding. 5 μm paraffin sections were melted briefly on a hot plate before the slides were dewaxed using xylene and then rehydrated using solutions of decreasing ethanol gradient (100–70%). The staining procedure was as follows: 5 min immersion in haematoxylin, 5 min in running water, 3–5 dips in acid alcohol, 2 min in running water, 5 dips in eosin and then 2 min in running water. After staining, the sections were dehydrated in an increasing ethanol gradient (70–100%). Finally, sections were immersed in xylene for 7 min before being mounted. Slides were imaged with a bright field channel on an Olympus BX40 microscope. A minimum of 60 islets per mouse (three mice/group) were scored for insulitis. Histological analysis of immune cell infiltrates in pancreatic islets was performed blinded by an independent observer (JK).

### Islet dissociation prior to flow cytometric analysis

Islets were isolated from MLDS mice, washed in PBS and dissociated with TrypLE Express (1 ml/100 islets) for 10 min at 37 °C, triturating every 5 min for 10 s. Samples were then washed with DMEM ( + 10% FBS) then PBS and transferred to round bottom tubes and kept on ice.

### Spleen dissociation and treatment prior to flow cytometric analysis

Mouse splenocytes were isolated as described in [42, 43]. On the day of sacrifice, spleens were removed, placed on ice in Hanks’ Balanced Salt Solution and mechanically disrupted using forceps. The cell suspensions were centrifuged at 1500 rpm (4 °C) for 5 min and erythrocytes were lysed by suspension of the pellets in 5 ml of ACK (Ammonium-Chloride-Potassium) Lysing Buffer (ThermoFisher Scientific, Altrincham, UK). Cell suspensions were then incubated at room temperature for 10 min, followed by centrifugation. Supernatants were discarded and pellets were washed twice with HBSS and resuspended in supplemented RPMI. An aliquot of cell suspension was mixed with 0.4% trypan blue solution (Sigma-Aldrich). Through the trypan blue exclusion method, cells were counted using a haemocytometer chamber. 1 × 106 cells/ml of cells were seeded on P24 round-bottom plates and cultured (37 °C; 5% CO_2_) in 10 µM EdU for 24 h. To detach from the flask, cells were then incubated (37 °C) in a solution of trypsin/EDTA (0.1%/0.02%) for 2 min, prior to addition of DMEM. After centrifugation at 1000 rpm for 2 min, the spleen cell pellet was re-suspended in fresh DMEM and kept on ice for flow cytofluorimetry analysis.

### Flow cytometric analysis

Single-cell suspensions were incubated with anti-mouse CD4, CD8 and F4/80 (all 1:50; to assess immune cell number), anti-mouse IFNγ or TNFα (both 1:50; immune cell activation) and anti-mouse insulin (1:100; beta-cell number) fluorochrome-conjugated monoclonal antibodies. Click-iT EdU Flow Cytometry Cell Proliferation Assay (Thermo Fisher Scientific, Hemel Hempstead, UK) was used to assess immune cell proliferation. See Supplementary Methods Gating strategies are shown in Figs. S5–S11.

### qPCR

Total mouse PDLN RNA was extracted using Trizol reagent (Invitrogen, Paisley, UK). Reverse transcription was performed using the High-Capacity cDNA reverse transcription kit (Applied Biosystems, Warrington, UK). Real-time qPCR was carried out with a LightCycler480, using Taqman methology (Quantitech, UK). Gene expression was measured by –ΔCt methodology, normalised against 18S (Quantitech, UK).

### Migration assays

Transwell CD8^+^T-cell migration assays were performed as previously described [60], using reformed islets originally isolated from BALB/C mice and haplotype-matched CD8^+^T-cells isolated from 8-week-old diabetic female NY8.3-NOD mice. Cells were exposed to pro-inflammatory cytokines ± FK866. After 18 h incubation, CD8^+^cell migration into islets was quantified by measuring the O.D values at Ex/Em = 540/590 nm using a PHERAstar Microplate Reader. See ‘Supplementary Methods’. Figure 6 shows a schematic representation of the experimental set-up.

### Data analysis

Significance was tested using one- or two-way ANOVA with Bonferroni’s or Tukey’s post-hoc test respectively, using GraphPad PRISM software (San Diego, CA, USA). Data are expressed as mean ± SEM.

## Supplementary information

Supplementary methods and data

## Data Availability

The datasets generated during the current study are available in the EMBL-EBI Array Express repository, https://www.ebi.ac.uk/biostudies/ArrayExpress/studies/E-MTAB-12382?query=E-MTAB-12382%20.
